# Supplementary figures and images for: Identifying and exploiting trait-relevant tissues with multiple functional annotations in genome-wide association studies
Source: PLoS Genet. 2018 Jan 29;14(1):e1007186. doi: 10.1371/journal.pgen.1007186 (PMC5805369; doi:10.1371/journal.pgen.1007186)

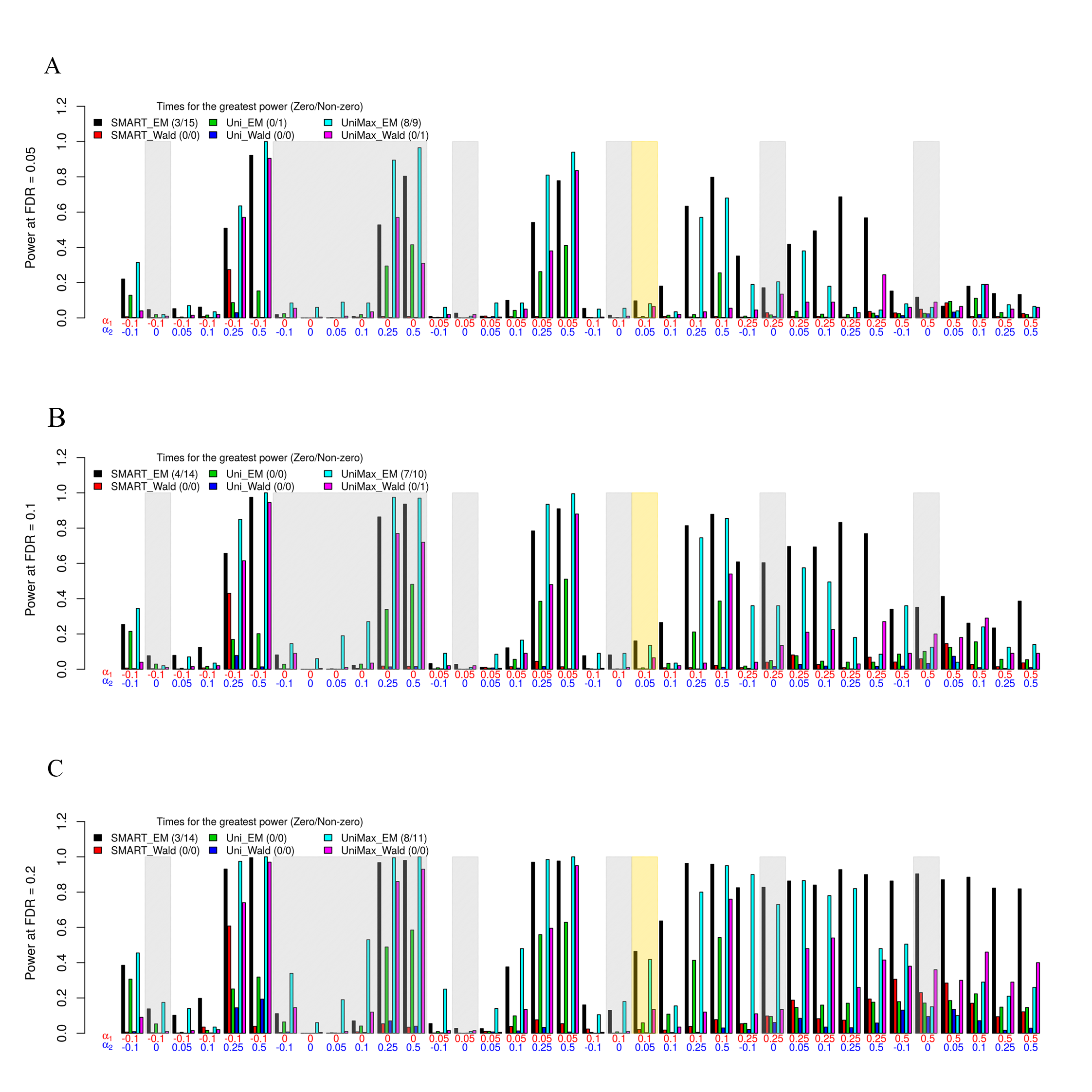

Supplement: S1 Fig — Power to detect trait-relevant tissues by different approaches in various settings at a fixed FDR of 0.05 (A), 0.1(B), or 0.2 (C). x-axis shows the values of the two annotation coefficients used in the simulations. Settings where at least one annotation coefficient is zero are shaded in grey. The setting where the annotation coefficients equal to the median estimates from real data (i.e. α = (0.1, 0.05)) is shaded in gold. (TIF) [file pgen.1007186.s001.tif]

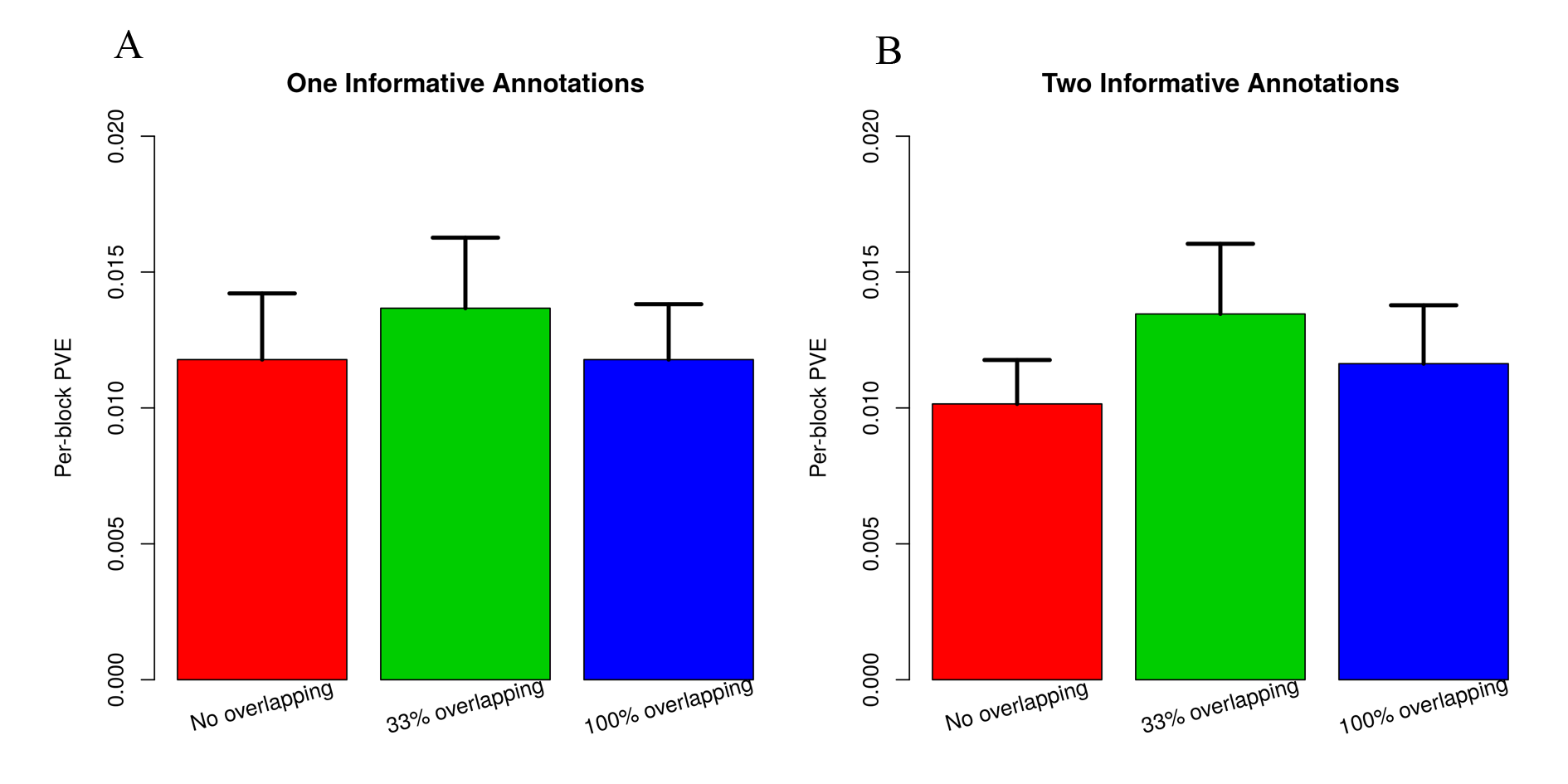

Supplement: S2 Fig — (A) The cases of one informative annotations at α0 = 0.5 and (α1, α2) = (0.4, 0); (B) The cases of two informative annotations at α0 = 0.5 and (α1, α2) = (0.4, 0.4). The bar indicates the standard error across simulation replicates. (TIF) [file pgen.1007186.s002.tif]

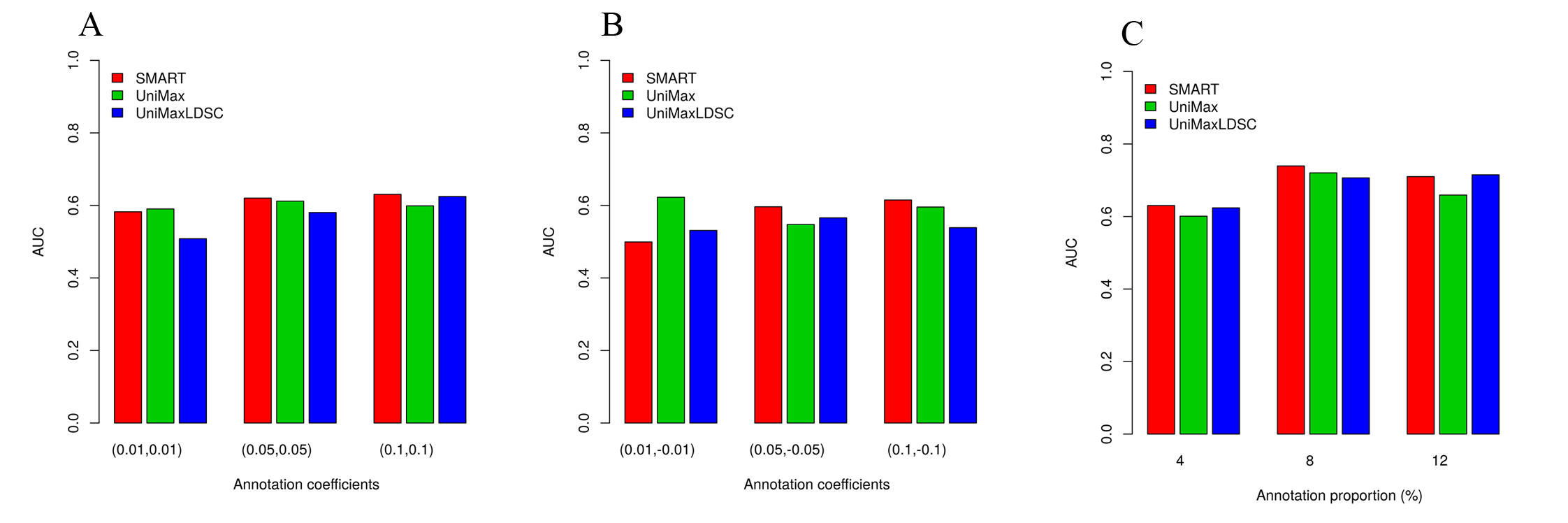

Supplement: S3 Fig — Methods for comparison include SMART (red), UniMax (green), and UniMaxLDSC (blue). Area under the curve (AUC) is used to measure method performance. (A) Power to identify trait-relevant tissue generally increases with increasingly large annotation coefficients when the two coefficients have the same sign. (B) Power also increases with increasingly large annotation coefficients when the two coefficients have the opposite sign. (C) Power is relatively stable with the genome coverage of the two annotations varied from 4%, 8% to 12%. (TIF) [file pgen.1007186.s003.tif]

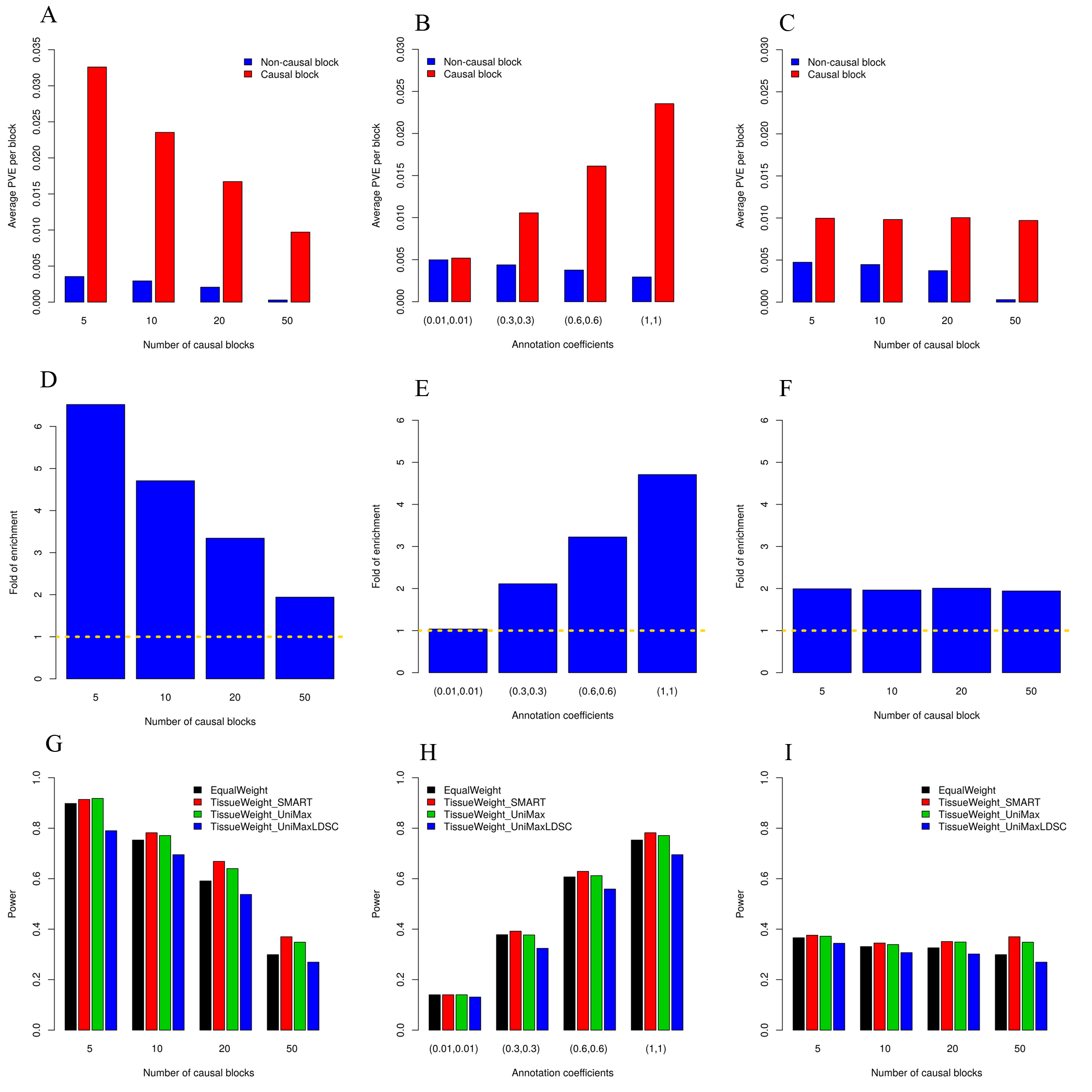

Supplement: S4 Fig — Left columns (A, D, G): annotation coefficients are fixed to be (1, 1) while the number of causal blocks changes from 5, 10, 20 to 50. Middle columns (B, E, H): the number of causal blocks is fixed to be 10 while the annotation coefficients change from (0.01, 0.01), (0.3, 0.3), (0.6, 0.6) to (1, 1). Right columns (C, F, I): per-block PVE are approximately fixed while the number of causal blocks and annotation coefficients vary. Top rows (A, B, C) show the average proportion of phenotype variance explained (PVE) by non-causal or causal blocks. Middle rows (D, E, F) show the fold enrichment. Bottom rows (G, H, I) show SNP set analysis power for various methods. (TIF) [file pgen.1007186.s004.tif]

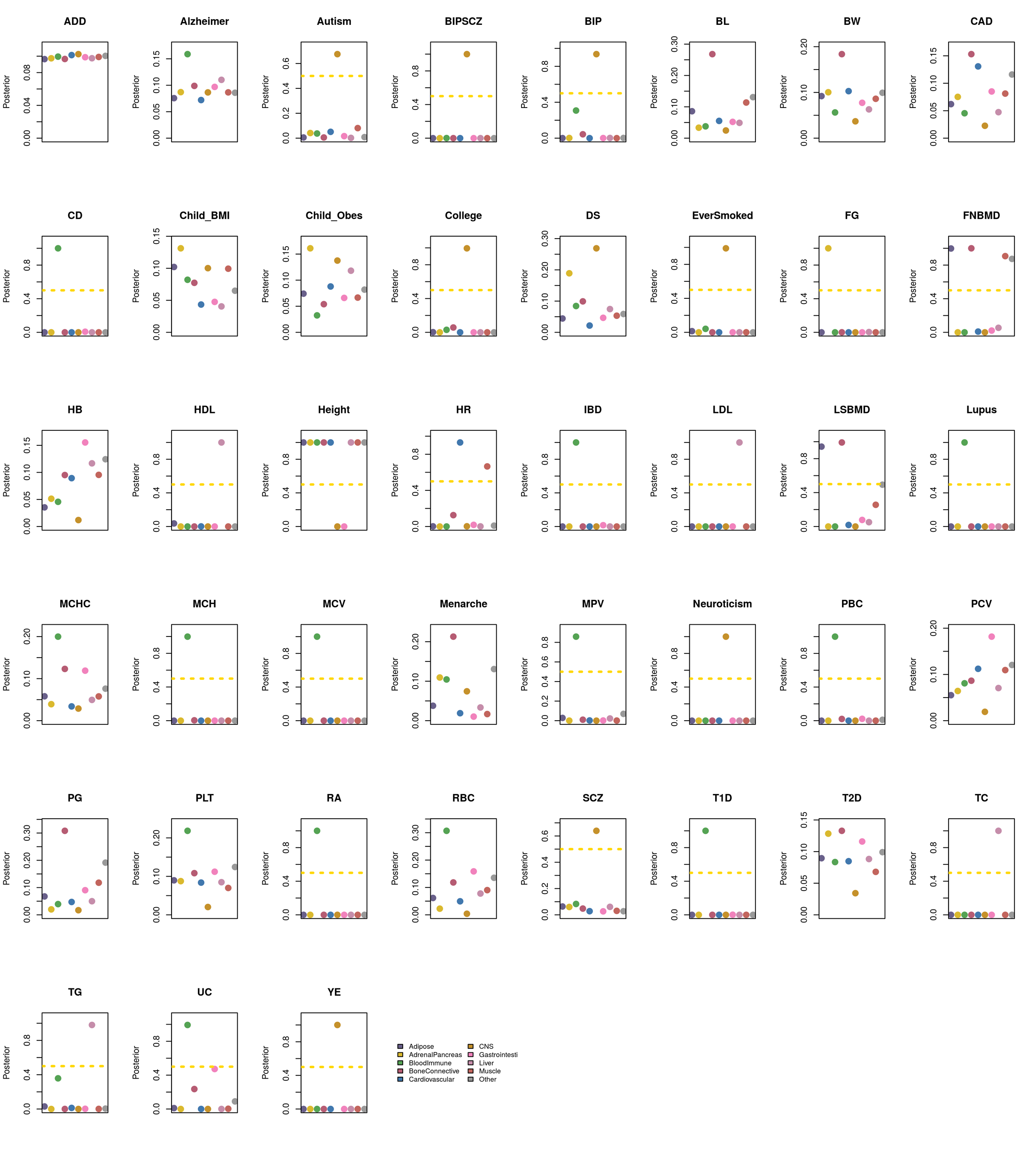

Supplement: S5 Fig — The gold dashed line represents a horizontal line at 0.5. (TIF) [file pgen.1007186.s005.tif]

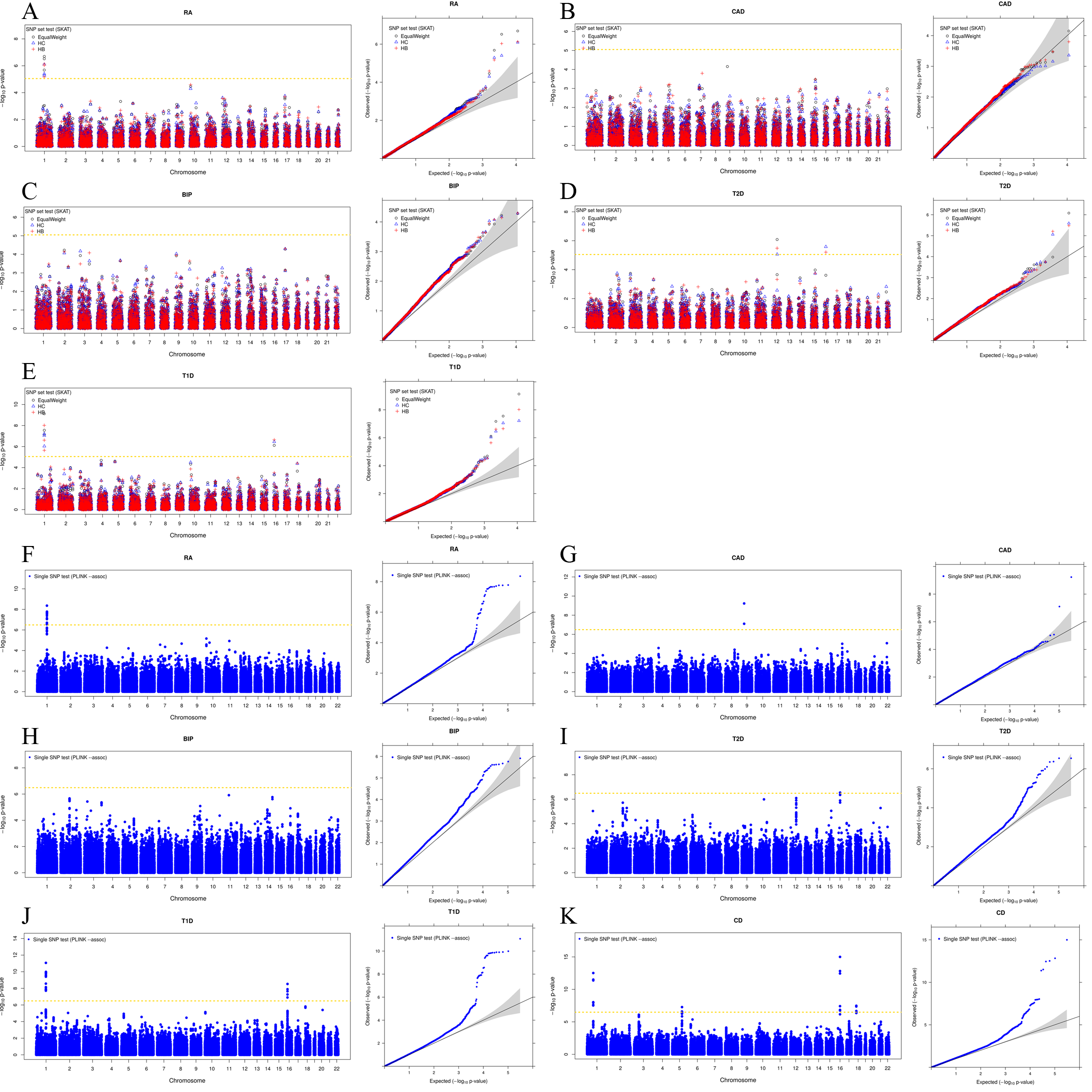

Supplement: S6 Fig — Results are shown for rheumatoid arthritis (RA; A), cardiovascular disease (CAD; B), bipolar disease (BIP; C), type II diabetes (T2D; D), and type I diabetes (T1D; E). For comparison, association results based on univariate SNP tests are also shown in F-K. EqualWeight (black): equal SNP weights. HC (blue): SNP weights constructed using the estimated coefficient parameters for continuous histone mark based annotations in a GWAS consortium study. HB (red): SNP weights constructed using the estimated coefficient parameters for binary histone mark based annotations in a GWAS consortium study. For Manhattan plots, gold dashed lines represent genome-wide significance thresholds: 0.05/153,813 for univariate tests and 0.05/5,588 for SNP set tests. For QQ plots, grey shaded area represents the 95% point-wise confidence interval. (TIF) [file pgen.1007186.s006.tif]

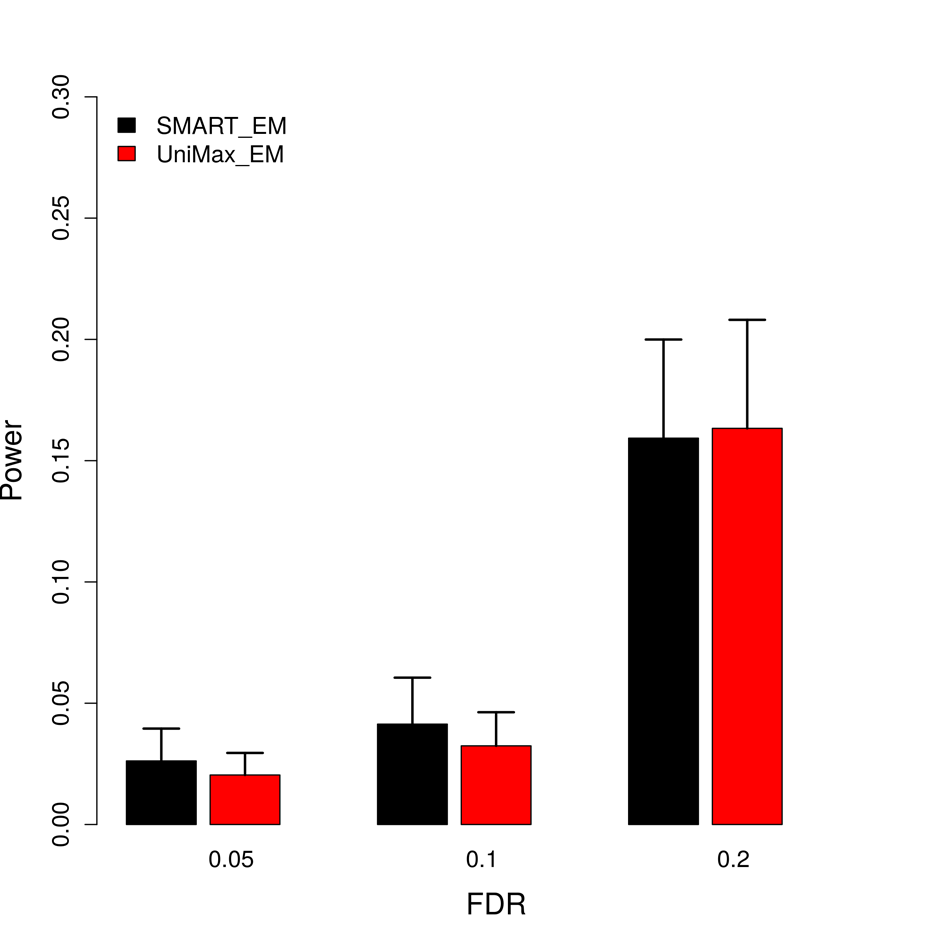

Supplement: S7 Fig — Among the ten tissues, we randomly selected two of them to be trait-relevant. We selected one annotation from the first trait-relevant tissue and the annotation from the second trait-relevant tissue to simulate SNP effects. The annotation coefficients for these two annotations are set to be 0.1 and 0.05, with α_0 = 0.1. We performed 5,000 simulation replicates that were divided into 10 groups. We computed power in each simulation group separately, and obtained the mean and standard deviation of power across 10 groups. The plot shows the mean power and its standard deviation to detect either of the two trait-relevant tissues by different approaches at a fixed FDR value of 0.05, 0.1 or 0.2. (TIF) [file pgen.1007186.s007.tif]
