# Supplementary material for: Identifying and exploiting trait-relevant tissues with multiple functional annotations in genome-wide association studies
Source: PLoS Genet. 2018 Jan 29;14(1):e1007186. doi: 10.1371/journal.pgen.1007186 (PMC5805369; doi:10.1371/journal.pgen.1007186)
Supplement: S1 Text — (DOCX) [file pgen.1007186.s013.docx]

**Method Details**

We consider the following linear regression model that links phenotypes to genotypes

|  | $\boldsymbol{y}=\boldsymbol{X\beta}+\boldsymbol{\epsilon}, \epsilon_{i}\sim N\left( 0, \sigma_{e}^{2} \right)$. | (1) |
| --- | --- | --- |

where ***y*** is an *n*-vector of phenotypes; $\boldsymbol{X}$ is an *n* by *m* matrix of genotypes; $\boldsymbol{\beta}$ is an *m*-vector of effect sizes; and $\boldsymbol{\epsilon}$ is an *n*-vector of residual errors and each $\epsilon_{i}$ follows an independent and identically distributed normal distribution with variance $\sigma_{e}^{2}$; and *n* is the sample size, *m* is the number of SNPs. We center the phenotype ***y*** and standardize each column of the genotype matrix $\boldsymbol{X}$ to have zero mean and unit variance, allowing us to ignore the intercept in the model.

For the *j*-th SNP, we denote $A_{j}=\left( 1,C_{j1}, C_{j2},\cdots,C_{jc} \right)^{T}$ as a (*c+*1)-vector of realized annotation values including a one that represents the intercept. These annotations can be either discrete or continuous. To simplify presentation, we assemble the annotation vectors across all SNPs into an *m* by (*c+*1) annotation matrix ***A***, where each row contains the annotation vector for the corresponding SNP

|  | $\boldsymbol{A}=\left[ \begin{matrix} {1 C}_{11} & \cdots& C_{1c} \\ \vdots& \ddots& \vdots\\ 1 C_{m1} & \cdots& C_{mc} \end{matrix} \right]$. | (2) |
| --- | --- | --- |

We assume that the effect size of each SNP $\beta_{j}$ is independent and follows a normal distribution with mean zero and a variance $\sigma_{j}^{2}$ that is SNP specific. We further impose an extra layer of hierarchy by assuming that the SNP specific variance $\sigma_{j}^{2}$is a function of the annotation vector

|  | $\beta_{j}\sim N\left( 0, {\sigma_{j}^{2}}/m \right) , \sigma_{j}^{2}=A_{j} \boldsymbol{\alpha}^{*}$, | (3) |
| --- | --- | --- |

where $\boldsymbol{\alpha}^{*}= \binom{\alpha_{0}}{\boldsymbol{\alpha}}$ is a (*c*+1)-vector of coefficients that include an intercept $\alpha_{0}$ and a *c*-vector of annotation coefficients $\boldsymbol{\alpha}$. It is reasonably assumed that the annotation coefficient $\boldsymbol{\alpha}$is large when the corresponding annotation is predictive of the SNP effect size. Therefore, the annotation coefficients can be used to evaluate the importance of annotations. Above, we center the second to the (c+1)-th columns of $A_{j}$ to have mean zero across SNPs.

Incorporating equation (3) into (1) leads to a joint model

|  | $\boldsymbol{y}\sim MVN\left( 0, \boldsymbol{H} \right), \boldsymbol{H}=\boldsymbol{X}\boldsymbol{D}_{\left( \boldsymbol{\alpha}^{\boldsymbol{*}} \right)}\boldsymbol{X}^{T}+ \sigma_{e}^{2}\mathbf{I}$, | (4) |
| --- | --- | --- |

where $\boldsymbol{D}_{\left( \boldsymbol{\alpha}^{\boldsymbol{*}} \right)}$ is an *m* by *m* diagonal matrix with $j^{\mathrm{th}}$ diagonal element $D_{\left( \alpha^{*} \right)jj}={\sigma_{j}^{2}}/m$, $\boldsymbol{H}$ is an *m* by *m* covariation matrix, and MVN denotes the multivariate normal distribution. Note that above we have assumed a linear relationship between $\sigma_{j}^{2}$ and the annotations $A_{j}$. While the linearity assumption does not always guarantee that each estimated variance ${\hat{\sigma_{j}}}^{2}$ is positive, the combined genetic variance $\boldsymbol{X}\boldsymbol{D}_{\left( \alpha^{*} \right)}\boldsymbol{X}^{T}$ in real data applications are always positive definite. We also acknowledge that we instead could have modeled a linear relationship between the log transformed variance and annotations (i.e. $\log\sigma_{j}^{2}=A_{j} \boldsymbol{\alpha}$) to ensure the positive value of the estimated ${\hat{\sigma_{j}}}^{2}$. However, we found that the log transformation of the variance made the inference algorithm unstable. Therefore, we use the simplified linear modeling assumption and set the estimated  ${\hat{\sigma_{j}}}^{2}$ to be zero in the rare cases when it is estimated to be negative.

Our goal is to infer the annotation coefficients $\boldsymbol{\alpha}^{*}$. To do so, we follow the main idea of LDSC [[1](#_ENREF_1)] and MQS [[2](#_ENREF_2)] in using the marginal $\chi^{2}$ statistics. Unlike the detailed algorithms of LDSC or MQS that were initially designed for a single binary annotation, however, we applied the generalized estimating equation (GEE) [[3](#_ENREF_3), [4](#_ENREF_4)] inference method that allows for the joint inference of multiple binary and continuous annotations. Specifically, we first obtain the marginal $\chi^{2}$ statistics for the $j^{\mathrm{th}}$ SNP as $\chi_{j}^{2}\approx\frac{\mathbf{y}^{T}X_{.j}X_{.j}^{T}\mathbf{y}}{n}$, where $X_{.j}$ is the $j^{\mathrm{th}}$ column of the genotype matrix and the approximation assumes small effect sizes – a property holds well in most GWASs. We can obtain the expectation of the marginal $\chi^{2}$ statistics as

|  | $E\left( \chi_{j}^{2} \right)=E\left( \frac{\mathbf{y}^{T}X_{.j}X_{.j}^{T}\mathbf{y}}{n} \right)=\frac{1}{n}tr\left( X_{.j}X_{.j}^{T}E\left( \boldsymbol{y}\boldsymbol{y}^{T} \right) \right)=\frac{1}{n}\sum_{i=1}^{m} \frac{X_{.i}^{T}X_{.j}X_{.j}^{T}X_{.i}\sigma_{i}^{2}}{m}+ \sigma_{e}^{2}$. | (5) |
| --- | --- | --- |

To simply notation, we denote ***R*** as an *m* by *m* correlation matrix $\boldsymbol{R}=\frac{\boldsymbol{X}^{T}\boldsymbol{X}}{n}$, $\boldsymbol{\Omega}=\boldsymbol{R} o \boldsymbol{R}$as an *m* by *m* LD matrix in the form of a Hadamard product between two $\boldsymbol{R}$ matrices (i.e. $\Omega_{ij}=R_{ij}^{2}$ for ${ij}^{\mathrm{th}}$ element), $1_{m}$ as an *m* vector of 1s, and $d_{\left( \alpha^{*} \right)}=\frac{\boldsymbol{A}\alpha^{*}}{m}$ as an *m* vector of the diagonal elements of $\boldsymbol{D}_{\left( \boldsymbol{\alpha}^{\boldsymbol{*}} \right)}$. We can express the *m*-vector $E\left( \chi^{2} \right)$as

|  | $E\left( \chi^{2} \right) =\left( \frac{n\boldsymbol{\Omega}\boldsymbol{A}}{m} ,1_{m} \right)\left( \begin{matrix} \alpha^{*} \\ \sigma_{e}^{2} \end{matrix} \right)=\boldsymbol{\Psi\Theta},$ | (6) |
| --- | --- | --- |

where we further denote $\boldsymbol{\Psi}=(\frac{n\boldsymbol{\Omega}\boldsymbol{A}}{m}, 1_{m})$ as the *m* by (*c+*2) design matrix and $\boldsymbol{\Theta}=\left( \begin{matrix} \alpha^{*} \\ \sigma_{e}^{2} \end{matrix} \right)$ as the (*c+*2)-vector of parameters.

With a heterogeneous error variance assumption, we set up the generalized estimating equation as

|  | $\boldsymbol{\Psi}^{T}\mathbf{W}\left( \chi^{2}-\boldsymbol{\Psi\Theta} \right)=0,$ | (7) |
| --- | --- | --- |

where **W** is an *m* by *m* diagonal working covariance matrix with $j^{\mathrm{th}}$ element $w_{j}$ that is directly taken from LDSC [[1](#_ENREF_1)]. In particular, $w_{j}=\frac{1}{{2l}_{j}\left( 1+\frac{nh^{2}\Sigma l_{jc}}{m} \right)^{2}}$, where $l_{j}=\sum\Omega_{j.}$ is the usual LD score for $j^{\mathrm{th}}$ SNP and $l_{jc}=\Omega_{j}.A_{.c}$ is the LD score for *j*^th^ SNP in the *c*^th^ annotation category, $h^{2}$ is the heritability equaling $\alpha_{0}$.

The above GEE equation leads to an iterative reweighted least squares method for estimating the parameters. After convergence, we obtain the estimates of $\boldsymbol{\Theta}$

|  | ${\hat{\boldsymbol{\Theta}}}^{(k+1)}=\left( \boldsymbol{\Psi}^{T}\mathbf{W}^{(k+1)}\boldsymbol{\Psi} \right)^{-}\boldsymbol{\Psi}^{T}{\mathbf{W}^{(k+1)}\chi}^{2}.$ | (8) |
| --- | --- | --- |

We use the robust sandwich estimator to obtain the covariance matrix $\mathrm{Cov}\left( \hat{\boldsymbol{\Theta}} \right)$ of $\hat{\boldsymbol{\Theta}}$. To do so, we recognize the covariance between two marginal $\chi^{2}$ statistics as

|  | $\mathrm{Cov}\left( \chi_{i}^{2}, \chi_{j}^{2} \right)=\frac{2}{n^{2}}tr\left[ X_{.i}X_{.i}^{T}HX_{.j}X_{.j}^{T}H \right]\approx\frac{2}{n^{2}}tr\left[ X_{.i}X_{.i}^{T}\boldsymbol{H}X_{.j}X_{.j}^{T}\boldsymbol{y}\boldsymbol{y}^{T} \right]$ | (9) |
| --- | --- | --- |
|  | $=\frac{2}{n}\frac{{\boldsymbol{y}^{T}X}_{.i}}{\sqrt{n}}X_{.i}^{T}\left( \boldsymbol{X}\boldsymbol{D}_{\left( \boldsymbol{\alpha}^{\boldsymbol{*}} \right)}\boldsymbol{X}^{\boldsymbol{T}}+ \sigma_{e}^{2}\mathbf{I} \right)X_{.j}\frac{X_{.j}^{T}\boldsymbol{y}}{\sqrt{n}}$, |  |

where the approximation is based on [[2](#_ENREF_2)]. Therefore, we have

|  | $Cov(\chi^{2})=2\boldsymbol{D}_{\boldsymbol{\chi}}(\frac{\boldsymbol{X}^{\boldsymbol{T}}\boldsymbol{X}\boldsymbol{D}_{\left( \boldsymbol{\alpha}^{\boldsymbol{*}} \right)}\boldsymbol{X}^{\boldsymbol{T}}\boldsymbol{X}}{n}+\frac{\boldsymbol{X}^{\boldsymbol{T}}\boldsymbol{X}\sigma_{e}^{2}}{n})D_{\chi}=2\boldsymbol{D}_{\boldsymbol{\chi}}(n\boldsymbol{R}\boldsymbol{D}_{\left( \boldsymbol{\alpha}^{\boldsymbol{*}} \right)}\boldsymbol{R}+\boldsymbol{R}\sigma_{e}^{2})\boldsymbol{D}_{\boldsymbol{\chi}},$ | (10) |
| --- | --- | --- |
|  | $\mathrm{Cov}\left( \hat{\Theta} \right)= \left( \boldsymbol{\Psi}^{\mathbf{T}}\boldsymbol{W\Psi} \right)^{\mathbf{-}}\boldsymbol{\Psi}^{\mathbf{T}}\mathbf{W}\mathrm{Cov}\left( \chi^{2} \right)\boldsymbol{W\Psi}\left( \boldsymbol{\Psi}^{\mathbf{T}}\boldsymbol{W\Psi} \right)^{\mathbf{-}}.$ | (11) |

where $\boldsymbol{D}_{\boldsymbol{\chi}}$ is an *m* by *m* diagonal matrix with j^th^ element $\sqrt{\chi_{j}^{2}}$.

With $\hat{\boldsymbol{\Theta}}$ and $\mathrm{Cov}\left( \hat{\boldsymbol{\Theta}} \right)$, we can extract the corresponding parts for the annotation coefficients from equations 8 and 11, and construct a Wald statistics as

|  | $h_{Wald}={\hat{\boldsymbol{\alpha}}}^{T}Cov\left( \hat{\boldsymbol{\alpha}} \right)^{-1}\hat{\alpha}.$ | (12) |
| --- | --- | --- |

Note that the LDSC paper used heritability enrichment for testing annotations but used z score of the coefficient directly for cell type-specific analyses. Therefore, above, we have followed the LDSC and did not use heritability enrichment for quantifying trait-tissue relevance. Using coefficients directly for trait-tissue relevance inference is preferred to using heritability enrichment as the former often provides more sensible results in practice. To illustrate this point, let’s consider a simple example where we have two functional annotations, each occupying an equal partition of the genome and each explaining 50% of heritability. In this case, there is no heritability enrichment for either annotation. However, if the two annotations from tissue A explain more heritability together than the two annotations from tissue B, while both occupying an equal proportion of the genome in the two tissues (i.e. similar standard errors for the annotation coefficients in the two tissues), then it seems natural to claim that tissue A is more relevant to the trait than tissue B. Therefore, we have followed LDSC to use Wald statistics on annotation coefficients directly in the present study for inferring trait-tissue relevance.

The GEE estimation procedure described above requires individual-level genotype data for the computation of the LD matrix $\boldsymbol{\Omega}$ and the correlation matrix **R**. When individual-level genotypes are not available, we can use a suitable reference panel for the computation of $\boldsymbol{\Omega}$ and **R**. In the present study, we used 503 individuals of European ancestry from the 1000 genomes project [[5](#_ENREF_5)] as the genotype reference panel. To further reduce computational cost and memory requirement, we followed [[6](#_ENREF_6)] and used a banded matrix plus a low rank matrix to approximate $\boldsymbol{\Omega}$ and **R** for each chromosome separately. In particular, we computed $\hat{\boldsymbol{\Omega}}$ and $\hat{\mathbf{R}}$ in the reference panel, extracted the banded parts ($\boldsymbol{\Omega}_{s} \mathrm{and} \mathbf{R}_{s}$) using a bandwidth of 1cM, and added a one-rank matrix ($\boldsymbol{\Omega}_{LR}$) with equal element 1/*n* to $\boldsymbol{\Omega}_{s}$ to ensure that the off-diagonal elements in the approximated$\boldsymbol{\Omega}$ matrix equal its expectation.

**Trait-Relevant Tissue Classification with EM**

Here, we present details for the expectation maximization (EM) algorithm that classifies tissues into two groups in terms of their trait-relevance. Specifically, we first compute the multivariate Wald statistics, $h_{t}$ for every tissue $t\in(1, \cdots,T)$. We then model these Wald statistics across tissues using a mixture of two non-central chi-squared distributions

|  | $h_{t} \sim\pi\chi_{\left( k, \lambda_{1} \right)}^{2}+\left( 1-\pi\right)\chi_{\left( k,\lambda_{0} \right)}^{2},$ | (13) |
| --- | --- | --- |

where, with proportion $\pi$, $h_{t}$ follows a chi-squared distribution with a large variance $\lambda_{1}$, while with proportion $1-\pi$, $h_{t}$ follows a chi-squared distribution with a small variance $\lambda_{0}$. Both chi-squared distributions share the same degrees of freedom $k$ that equals to the number of annotations used in the Wald statistics (i.e. *c*). However, the two distribution have different noncentrality parameters $\lambda_{1}$ and $\lambda_{0}$ with $\lambda_{1}>\lambda_{0}$. The chi-squared distribution with the small noncentrality parameter represents the empirical null distribution that contains tissues irrelevant to the trait. The small, nonzero, noncentrality parameter characterizes the fact that these irrelevant tissues tend to have Wald statistics larger than what would be expected under the theoretical null distribution (i.e. central chi-squared) simply due to annotation correlation across tissues. In contrast, the chi-squared distribution with the large non-central parameter represents the alternative model that contains tissues relevant to the trait. The large noncentrality parameter characterizes the fact that these relevant tissues tend to have Wald statistics larger than those from the irrelevant tissues. To complete the model specification, we specify a beta prior for $\pi$, where we set the first shape parameter $b_{1}$ to be the number of tissues and the second shape parameter $b_{2}$ to be nine times the first so that the prior expectation of $\pi$ is 0.1 with the belief that only a fraction of tissues are related to the given trait.

We use the EM algorithm to infer λ_1_, λ_0_ and π. To facilitate inference, we introduce a vector of latent variables $z_{t}$ that equals 1 if $h_{t}$ follows the alternative distribution and equals 0 if $h_{t}$ follows the null distribution. Our goal is thus to infer the posterior probability (PP) of each tissue that belongs to the first component, or $P(z_{t}=1)$.

In the EM algorithm, the expectation (E)-step is

|  | $\pi_{t}^{\left( s \right)}=\frac{\pi^{\left( s \right)}P\left( h_{t} \vert\lambda_{1}^{\left( s \right)},k \right)}{\pi^{\left( k \right)}P\left( h_{t} \vert\lambda_{1}^{\left( s \right)},k \right)+(1- \pi^{\left( s \right)})P\left( h_{t} \vert\lambda_{0}^{\left( s \right)},k \right)}.$ | (14) |
| --- | --- | --- |

While the maximization (M)-step is

|  | ${(\lambda}_{1}^{\left( s+1 \right)}, \lambda_{0}^{\left( s+1 \right)},\pi^{\left( s+1 \right)})$ | (15) |
| --- | --- | --- |
|  | $=argmax\left( Q \right)$ |  |
|  | $=argmax\left\{ \Sigma\left[ \left( log\left( \pi\right)+\log\left( P\left( h_{t} \vert\lambda_{1},k \right) \right) \right)\pi_{t}^{\left( s \right)}+\left( log\left( 1-\pi\right)+\log\left( P\left( h_{t} \vert\lambda_{1},k \right) \right) \right)(1-\pi_{t}^{\left( s \right)}) \right]+\left( b_{1}-1 \right) log\left( \pi\right)+\left( b_{2}-1 \right)log\left( 1-\pi\right) \right\}$ |  |
|  | $=argmax\left\{ log\left( \pi\right)*\left( \Sigma\pi_{t}^{\left( s \right)}+ b_{1}-1 \right) \right.+ log\left( 1-\pi\right)*\left( \Sigma\left( 1-\pi_{t}^{\left( s \right)} \right)+ b_{2}-1 \right)+ \Sigma\pi_{t}^{\left( s \right)}\log\left( P\left( h_{t} \vert\lambda_{1},k \right) \right)\left. + \Sigma(1-\pi_{t}^{\left( s \right)})\log\left( P\left( h_{t} \vert\lambda_{0},k \right) \right) \right\}$ |  |

We iterate between the E-step and M-step until convergence; the convergence criterion was defined as the absolute difference between two consecutive values for the likelihood is smaller than 0.001.

**Additional Simulation Details and Results**

We present part of the results from the first set of simulations described in the Materials and Methods here to illustrate the benefits of using mixture models to post-process the Wald statistics in order to address correlations among annotations and reduce false positives. To do so, we consider six different approaches:

(1) SMART_Wald. We analyzed two annotations jointly and computed a single multivariate Wald statistic for each tissue using our procedure. We used these Wald statistics to measure trait-tissue relevance.

(2) SMART_EM. We applied an EM algorithm and a mixture model on the multivariate Wald statistics computed in (1) to further classify tissues into two groups. We used the posterior probability of a tissue being trait-relevant to measure trait-tissue relevance.

(3) Uni_Wald. We analyzed one annotation at a time and computed two univariate Wald statistics for each tissue using our procedure. We used these Wald statistics to measure trait-tissue relevance.

(4) Uni_EM. On top of (3), we applied an EM algorithm to classify these Wald statistics into two groups. For each tissue and each annotation, we obtained the posterior probability of being a trait-relevant tissue to measure trait-tissue relevance.

(5) UniMax_Wald. We analyzed one annotation at a time. For each tissue, we computed two univariate Wald statistic using our procedure and selected among them the larger statistic as a measurement of trait-tissue relevance.

(6) UniMax_EM. On top of (5), we applied an EM algorithm to classify these Wald statistics into two groups. For each tissue, we obtained the posterior probability of its being a trait-relevant tissue to measure trait-tissue relevance.

We considered a range of realistic annotation coefficient combinations (i.e. $(\alpha_{1}, \alpha_{2})$). For each combination, we performed 1,000 simulation replicates. For each method, we computed the power of various methods in detecting the trait-relevant tissue at a false discovery rate (FDR) of 0.05, 0.1 or 0.2 (Figure S1). As mentioned in the Methods section, we recommend using an EM algorithm and a mixture model to post-process the Wald statistics in order to address correlations among annotations and reduce false positives. Indeed, using mixture modeling for post processing (i.e. SMART_EM, Uni_EM, and UniMax_EM) almost always results in better performance than using the raw Wald statistics alone (i.e. the corresponding SMART_Wald, Uni_Wald, and UniMax_Wald). We extract a subset of Figure S1 to be Figure 1A and present the results in the main text to compare a multivariate method (2) versus two univariate methods (4 and 6).

For simulation results presented in Supplementary Figure S3, we aim to explore the characteristics of annotations that can influence the power of SMART in identifying trait-relevant tissues. To do so, we simulated annotations that have various genome-occupancy characteristics and that have various annotation effect sizes and signs. Specifically, we simulated two binary annotations for each of the ten tissues, and each annotation annotates a fixed percentage of total SNPs to have value one and annotates the rest of SNPs to have value zero. We denote this fixed percentage as genome coverage, which varies from 4%, 8% to 12%. We set the overlap proportion among annotations in the trait-relevant tissue and trait-irrelevant tissues so that we can induce a correlation among annotations across tissues to be 0.5, a value close to that estimated in the real data. With these synthetic annotations, we then used 10,000 individuals and 10,000 SNPs on chromosome one from the GERA study and simulated phenotypes, in a similar fashion as those described in the first set of simulations in Materials and Methods. We considered three approaches SMART, UniMax and UniMax_LDSC as described in the main text. We considered three simulating settings where each setting examines one characteristic of the annotations:

(1) We fixed the genome-coverage of the annotations to be 4% while varied the annotation coefficients for the two annotations in the trait-relevant tissue to be (0.01, 0.01), (0.05, 0.05) or (0.1, 0.1);

(2) We fixed the genome-coverage of the annotations to be 4% while varied the annotation coefficients for the two annotations in the trait-relevant tissue to be (0.01, -0.01), (0.05, -0.05) or (0.1, -0.1);

(3) We fixed the annotation coefficients for the two annotations in the trait-relevant tissue to be (0.1, 0.1) while changed the genome-coverage of the annotations to be 4%, 8% or 12%;

In each simulation setting, we performed 1,000 simulation replicates, combined results across replicates, and computed the area under the curve (AUC) to compare the performance of different methods.

For simulation results presented in Supplementary Figure S4, we used 10,000 individuals and 10,000 SNPs from the GERA study and simulated phenotypes in a similar fashion as the second set of the simulations described in Materials and Methods. Briefly, we divided SNPs into 100 blocks with 100 SNPs in each block. We then simulated two binary annotations for each of the ten tissues, where each of the two annotations in the causal blocks of the trait-relevant tissue labels a random set of 40% SNPs to have value one and the rest SNPs to have value zero. For trait-irrelevant tissues, a same number of SNPs were annotated randomly to have annotation value of one. For the trait-relevant tissue, only SNPs inside the causal blocks may have annotation value of one, so the fold of the enrichment (**fe**) for the annotations is proportional to the per causal block PVE, where $fe=\frac{\mathrm{prop}\left( \mathrm{PVE}_{\mathrm{casal}} \right)}{prop\left( SNP_{causal} \right)}=\frac{\frac{N_{causalblock}PVE_{perCausal}}{h_{sim}^{2}}}{\frac{N_{block}}{N_{causalblock}}}=\frac{PVE_{perCausal}}{h_{sim}^{2}/N_{block}}$. We then performed weighted SKAT analysis using weights inferred by SMART_EM, UniMax_EM and UniMax_LDSC were applied. For UniMax_LDSC, 75 baseline annotations were used to address the correlation among annotations, and when computed the SNP specific variance as weights, the baseline annotations were not included:

(1) We fixed the annotation coefficients to be (1, 1) and varied the number of causal blocks to be 5, 10, 20 or 50;

(2) We fixed the number of causal blocks to be 10 and varied the annotation coefficients to be (0.01, 0.01), (0.3, 0.3), (0.6, 0.6) or (1, 1);

(3) We fixed the per-block PVE to be 0.1, and changed the number of causal blocks and annotation coefficients.

For each simulation scenario, 100 simulation replicates were performed.

**References**

1. Finucane HK, Bulik-Sullivan B, Gusev A, Trynka G, Reshef Y, Loh P-R, Anttila V, Xu H, Zang C, Farh K: **Partitioning heritability by functional annotation using genome-wide association summary statistics**. *Nature genetics* 2015, **47**(11):1228-1235.

2. Zhou X: **A unified framework for variance component estimation with summary statistics in genome-wide association studies**. *bioRxiv* 2016:042846.

3. Liang K-Y, Zeger SL: **Longitudinal data analysis using generalized linear models**. *Biometrika* 1986:13-22.

4. Chen WM, Broman KW, Liang KY: **Quantitative trait linkage analysis by generalized estimating equations: unification of variance components and Haseman-Elston regression**. *Genetic epidemiology* 2004, **26**(4):265-272.

5. Consortium GP: **An integrated map of genetic variation from 1,092 human genomes**. *Nature* 2012, **491**(7422):56-65.

6. Wen X, Stephens M: **Using linear predictors to impute allele frequencies from summary or pooled genotype data**. *The annals of applied statistics* 2010, **4**(3):1158.
